# Mitochondrial aggregation caused by cytochalasin B compromises the efficiency and safety of three-parent embryo

**DOI:** 10.1093/molehr/gaac036

**Published:** 2022-10-20

**Authors:** Ying Li, Sanbao Shi, Jin Yuan, Xi Xiao, Dongmei Ji, Jianxin Pan, Zhunyuan Min, Hao Wang, Hongying Sha, Yazhong Ji

**Affiliations:** Reproductive Medicine Center, Tongji Hospital Affiliated to Tongji University, Shanghai, China; State Key Laboratory of Medical Neurobiology and MOE Frontiers Center for Brain Science, Institutes of Brain Science, Fudan University, Shanghai, China; Department of Obstetrics and Gynecology, Center for Reproductive Medicine, The First Hospital Affiliated for Anhui Medical University, Hefei, China; The International Peace Maternal and Child Health Hospital, School of Medicine, Shanghai Jiao Tong University, Shanghai, China; Reproductive Medicine Center, Tongji Hospital Affiliated to Tongji University, Shanghai, China; Department of Obstetrics and Gynecology, Center for Reproductive Medicine, The First Hospital Affiliated for Anhui Medical University, Hefei, China; State Key Laboratory of Medical Neurobiology and MOE Frontiers Center for Brain Science, Institutes of Brain Science, Fudan University, Shanghai, China; State Key Laboratory of Medical Neurobiology and MOE Frontiers Center for Brain Science, Institutes of Brain Science, Fudan University, Shanghai, China; State Key Laboratory of Medical Neurobiology and MOE Frontiers Center for Brain Science, Institutes of Brain Science, Fudan University, Shanghai, China; State Key Laboratory of Medical Neurobiology and MOE Frontiers Center for Brain Science, Institutes of Brain Science, Fudan University, Shanghai, China; Reproductive Medicine Center, Tongji Hospital Affiliated to Tongji University, Shanghai, China

**Keywords:** mitochondrial replacement, germ cells, oocyte, cytochalasin B, mtDNA heteroplasmy

## Abstract

It is widely accepted that cytochalasin B (CB) is required in enucleation of the oocyte in order to stabilize the cytoplasm. However, CB treatment results in the uneven distribution of mitochondria, with aggregation towards the nucleus, which might compromise the efficiency and safety of a three-parent embryo. Here, we demonstrated that CB treatment affected mitochondrial dynamics, spindle morphology and mitochondrial DNA carryover in a concentration-dependent manner. Our results showed that mouse oocytes treated with over 1 μg/ml CB exhibited a more aggregated pattern of mitochondria and diminished filamentous actin expression. Abnormal fission of mitochondria together with changes in spindle morphology increased as CB concentration escalated. Based on the results of mouse experiments, we further revealed the practical value of these findings in human oocytes. Chip-based digital PCR and pyrosequencing revealed that the mitochondrial carryover in reconstituted human embryos was significantly reduced by modifying the concentration of CB from the standard 5 μg/ml to 1 μg/ml before spindle transfer and pronuclear transfer. In conclusion, our findings provide an optimal manipulation for improving the efficiency and safety of mitochondrial replacement therapy.

## Introduction

Mitochondrial replacement therapies or techniques (MRT) hold immense promise to avoid transmission between generations of mitochondrial DNA (mtDNA) mutations, which are maternally inherited and associated with incurably devastating disorders or fatal diseases (Wolf *et al.*, 2015). The severity of inherited mitochondrial disease symptoms depends on the ratio of mutant to wild-type mtDNA inside the cell and tissue, hereinafter referred to as heteroplasmy level (Tachibana *et al.*, 2013). In recent reports, 9.23% of mutant mtDNA is detected in the circumcised foreskin of a live birth baby derived from a three-parent human embryo (Zhang *et al.*, 2017) and, unexpectedly, reversion back to the mutant mtDNA genotype have been observed by three laboratories in up to 15% of the embryonic stem cells derived from human embryos after MRT (Hyslop *et al.*, 2016; Kang *et al.*, 2016; Yamada *et al.*, 2016; Chinnery, 2020). These findings arouse safety considerations regarding MRT and highlight the importance of reducing carryover to the lowest possible level. A number of technical and biological attempts have been made to minimize carryover of mtDNA, including refinements in mechanical micromanipulation, mitophagy, polar body transfer, genome editing, oocytes generated *in vitro* and cytoskeletal disruption (Wang *et al.*, 2014; Hyslop *et al.*, 2016; ZHou *et al.*, 2016; Greenfield *et al.*, 2017; Gammage *et al.*, 2018). For cytoskeletal disruption, a number of studies have been conducted, but seldom on cytochalasin B (CB) concentration. In the process of MRT, application of CB is required during enucleation to stabilize the cytoplasm (Hou *et al.*, 2016). However, the effects of CB on the efficiency and safety of mitochondrial displacement are poorly understood with no systematic experimental data.

It has been suggested that the application of CB in MRT is beneficial for reducing mtDNA carryover in spindle transfer (ST) and pronuclear transfer (PNT). One important reason for the likelihood of carryover in MRT is that mitochondria are connected to each other and to other organelles (Katayama *et al.*, 2006; Shoji *et al.*, 2006). Some studies suggest that a meshwork of cytoplasmic filamentous actin (F-actin) surrounds the meiotic spindle in maturing mouse oocytes (Azoury *et al.*, 2008, 2011), and an actin network is also observed around pronuclei in mouse zygotes (Chew *et al.*, 2012; Chaigne *et al.*, 2016; Okuno *et al.*, 2020). It has also been reported that a contractile nuclear actin network drives the chromosome congression in starfish oocytes (Lenart *et al.*, 2005) and a network of sub-oolemmal actin is displayed in human mature oocytes (Coticchio *et al.*, 2014). The F-actin network plays an important role in maintaining the spindle position for meiosis and mitosis. Since cell division requires an energy supply, there is a high possibility that these F-actin networks would entrap mitochondria (Greenfield *et al.*, 2017). In this case, CB inhibits F-actin polymerization thereby leading to reduced carryover in ST and PNT.

Nevertheless, another general scenario instead proposes that CB could result in higher mtDNA carryover in ST and PNT that might be related to the effects of CB on mitochondrial fission and cytoskeletal disorder. Mitochondrial shape and size are controlled by precisely regulated rates of fusion and fission (Van Blerkom, 2011). An imbalance of these two processes can dramatically alter mitochondrial morphology and the overall network structure (Chen and Chan, 2005). Recent studies indicate that F-actin transiently assembling on the outer mitochondrial membrane contributes to mitochondrial fission and that, vice versa, down-regulation of actin regulatory proteins induces fusion and leads to elongation of mitochondria (Li *et al.*, 2015). Moreover, actin filaments are heavily involved in cytokinesis, mitochondrial clustering and spindle positioning in mouse oocytes (Coticchio *et al.*, 2015; Moore *et al.*, 2016; Duan *et al.*, 2020). A study reports that mitochondria aggregate at the perinuclear region in CB-treated oocytes after germinal vesicle (GV) breakdown through some cytoskeleton-dependent mechanisms (Takahashi *et al.*, 2016). These reports indicate that homeostasis of the mitochondrial network could be disturbed by CB, leading to high heteroplasmy in ST and PNT.

Thus, in this article, we focus on the action of CB on the efficiency and safety of MRT. We investigated the effects of different concentrations of CB on mitochondrial distribution and morphology in both mouse and human oocytes, and also explored the possible underlying mechanism(s), aiming to find the optimal concentration of CB to minimize mtDNA carryover in reconstructed embryos after MRT.

## Materials and methods

### Reagents and media

The media used for the manipulation and culturing of mouse and human oocytes/zygotes were from Vitrolife Sweden AB. CB (C6762, Sigma, Saint Louis, MO, USA) was dissolved to a stock concentration of 10 mg/ml in dimethyl sulfoxide (DMSO, D2650, Sigma). Then the CB stock solution was diluted to gradient concentrations with G-GAMETE (10126, Vitrolife, Göteborg, Sweden). An additional amount of DMSO was added such that the final concentration of DMSO in each CB group was 0.1%. The 0 μg/ml CB group (DMSO group) represented zero CB + 0.1% DMSO, the blank group represented zero CB + 0% DMSO.

### Ethics statement

This study, conducted on human oocytes, was approved by the ethics committee of Tongji University (2014yxy10) and the First Affiliated Hospital of Anhui Medical University (20160022). All donor patients received a full explanation of the experiments and provided signed informed consent. The BDF1 (C57/BL6 × DBA) mice used in this study were maintained in accordance with the guidelines of the Shanghai Medical College, Fudan University (20160225-103).

### Human oocyte collection and IVM

The human immature oocytes were donated by patients who received IVF treatment for oviductal or male factors in Tongji Hospital Affiliated to Tongji University and the First Affiliated Hospital of Anhui Medical University. Since maternal age impacts the quality and shape of the oocyte mitochondria (May-Panloup *et al.*, 2016), as well as the structure and abundance of actin cytoskeleton (Coticchio *et al.*, 2014; Limatola *et al.*, 2019), the human oocyte donors enrolled in this study were all young women at childbearing age, between 25 and 33 years old (mean ± SD: 27.9 ± 2.2) to minimize the possible effects caused by age. Patients underwent controlled ovarian stimulation using a standard long pituitary down-regulation protocol with a GnRH agonist (GnRHa). In our centers, fertilization was checked 4.5 h after IVF, then unfertilized oocytes (at GV or metaphase I (MI) stage) were matured to metaphase II (MII) stage at 37°C under conditions of 6% CO_2_ and 5% O_2_ in a defined IVM medium, which was composed of M199 medium (Sigma) supplemented with 20% fetal bovine serum (Gibco, Thermo Fisher Scientific, New York, USA), 0.6 g/l penicillin (Sigma), 0.6 g/l streptomycin (Sigma), 0.5 IU/ml hCG (Livzon, Zhuhai, China), 0.1 mg/ml 17 β-estradiol (Sigma), 0.075 IU/ml FSH (Gonal-F, Merck Serono, Darmstadt, Germany), 0.22 mM pyruvic acid (Sigma) and 10 μM melatonin (Sigma), as described in previous studies (Zou *et al.*, 2020; Zhang *et al.*, 2021). Extrusion of the first polar body was used as the criterion for nuclear maturation. Patients with an average 13.6 ± 2.8 oocytes retrieved and more than 60% rate of fertilization donated 1–3 IVM-MII oocytes for this study. Eighty-five IVM-MII oocytes (from 41 donors, average 2.1 ± 0.6 IVM-MII oocytes from each donor) were donated for this study.

### Mouse oocyte collection

BDF1 female mice (6–8 weeks old) were superovulated by injection of 5 IU PMSG and 5 IU hCG. Oocyte–cumulus complexes were released 14 h after hCG and then incubated in hyaluronidase to denude the cumulus. MII oocytes (n = 1569) were divided into the blank group, the DMSO group (0 μg/ml group, control group) and the experimental groups. The blank group was cultured in G-GAMETE medium. The DMSO group was cultured in G-GAMETE medium + 0.1% DMSO, the experimental groups were cultured in G-GAMETE medium + 0.1% DMSO supplemented with different concentrations of CB (0.5, 1, 2.5, 5 and 10 μg/ml) for 30 min and then the oocytes were washed in G-GAMETE medium.

### Mitochondrial distribution

A total of 1139 mouse MII oocytes were incubated in 250 nM MitoTracker Red (M7512, Life Technologies, Thermo Fisher Scientific, Eugene, OR, USA) and 1 mg/ml Hoechst 33342 (R37605, Life Technologies, Thermo Fisher Scientific) for 20 min. Subsequently, oocytes were thoroughly rinsed at room temperature for a total of 10 min (3, 3 and 4 min, successively) in 1 ml of G-GAMETE medium. Afterwards, the stained oocytes were allocated into five experimental groups, a DMSO and a blank group, and cultured for 30 min. Three biological replicates were set up in each group, and the oocyte numbers are shown in Supplementary Table SI. Under a confocal microscope equipped with Lecia Confocal Software (SP2) (Leica Microsystems, Inc., Wetzlar, Germany), mitochondrial distributions in oocytes were observed and evaluated according to the criteria, which are adapted from previous studies (Liu *et al.*, 2010; Cao *et al.*, 2017; Stimpfel *et al.*, 2017): an aggregated pattern (Pattern A) is defined as over 50% of the mitochondria aggregated around the chromosomes; a dispersive pattern (Pattern D) means that mitochondria are dispersed throughout the cytoplasm. The evaluation was performed in a blinded manner by two independent well trained investigators. The percentage of oocytes in Pattern A (%) was calculated by the formula: [(oocyte number labeled pattern A by observer 1 + oocyte number labeled Pattern A by observer 2)/total oocyte number × 2] × 100.

### Mouse spindle–chromosomal complex transfer and IVF

The mouse ST procedure was performed as previously described (Tachibana *et al.*, 2010; Wang *et al.*, 2014). In brief, MII oocytes were placed into separate 10 μl droplets of G-GAMETE medium containing CB in a glass-bottom dish. The dish was placed on the stage of an inverted microscope (Nikon TE 2000S, Tokyo, Japan) equipped with stage warmer (Tokai Hit, Fujinomiya, Japan), Narishige micromanipulators and Oosight imaging software (Hamilton Thorne, Beverly, MA, USA). One oocyte was secured by the holding pipette, and rotated until the spindle was situated at the 3 o’clock position. The zona pellucida next to the spindle was pierced through carefully, with a sharp needle, to make a hole. Then, an enucleation pipette of 20 mm diameter was inserted through the opening. The spindle was gently aspirated into the pipette with a minimal amount of cytoplasm and then briefly exposed to the HVJ-E (inactivated Hemagglutinating Virus of Japan envelope, Genome One, Cosmo Bio, Tokyo, Japan) drop and released into the perivitelline space of another enucleated recipient oocyte, opposite to the first polar body. The reconstructed oocytes were left in the manipulation drop for 10–20 min until fusion occurred.

IVF was conducted after the reconstructed oocytes were rinsed in HTF medium several times and placed in HTF medium. Spermatozoa were obtained from the cauda epididymides of BDF1 males (10–12 weeks old) and transferred into the center of 200 μl HTF medium droplets for 1 h, for capacitation. The sperm suspension was used for fertilization at a final concentration of 2 × 10^6^/ml. The sperm and reconstructed oocytes were co-cultured in HTF medium at 37.5°C for 6 h. Oocytes developing two visible pronuclei (2PN) were considered to be fertilized and transferred into 100 ml G-1 PLUS (10128, Vitrolife, Göteborg, Sweden) medium and cultured up to 120 h at 37.5°C under 5% CO_2_, 5% O_2_ and 90% N_2_ incubation.

### Mouse pronucleus transfer

Mouse zygotes were placed in enucleation medium with CB. The zona pellucida penetration of both donor and recipient zygotes was performed as mentioned above. Then karyoplasts containing the 2PN of a recipient zygote were enucleated. Donor karyoplasts were isolated and aspirated in a pipette with a diameter of 15 mm. After brief exposure to HVJ-E, donor karyoplasts were gently expelled into enucleated recipient zygotes and allowed to fuse with the cytoplast. The reconstructed zygotes were washed three times in G-1 PLUS medium, and then were cultured in G-1 PLUS medium at 37.5°C, in a gas mixture of 5% CO_2_, 5% O_2_ and 90% N_2_ for 72 h.

### Immunofluorescence of mouse oocytes

MII oocytes were fixed in 4% paraformaldehyde containing 0.01% Triton X-100 for 30 min, followed by permeabilization and blocking in 0.1% Triton X-100 and 10% donkey serum for 2 h at room temperature. Then, the oocytes were incubated with primary antibody (anti-α-tubulin-FITC, F2168, Sigma, 1:500) overnight at 4°C. After washing in PBS three times, these oocytes were counterstained with rhodamine phalloidin (R415, Thermo Fisher, Eugene, OR, USA) for 2 h at room temperature. After being washed with PBS, oocytes were stained with Hoechst 33342 for 20 min and then analyzed under a confocal microscope.

### Transmission electron microscopy

To assess the mitochondrial ultrastructure in oocytes, mouse MII oocytes from the 0, 1 and 5 μg/ml CB groups were fixed in 2.5% glutaraldehyde at 4°C for 2 h. Then oocytes were stained with eosin for 15 min and embedded in 1.2% agarose. Then the agarose gel cube was fixed in 2.5% glutaraldehyde overnight at 4°C. The specimens were washed with 0.1 M phosphate buffer (PB), postfixed in 1% osmium tetroxide for 1 h and washed with 0.1 M PB. Samples were then dehydrated in an ascending series of ethanol dilutions and washed three times in 100% acetone. They were washed with a mixture of 50% acetone and 50% resin for 3 h and then infiltrated in resin overnight. Ultrathin sections (70 nm) were stained with uranyl acetate and lead citrate, then examined using transmission electron microscopy (TEM) (JEM-2100; JEOL, Tokyo, Japan) with TEMography™ software (JEOL, Tokyo, Japan).

### Oocyte spindle imaging

Live imaging was performed using the Oosight imaging system (Hamilton Thorne, Beverly, MA, USA) to detect the effect of CB on the spindle shape. Samples (mouse and human oocytes, human karyoplasts) were transferred into G-GAMETE droplets covered with mineral oil in a 29 mm glass-bottom dish, which was loaded onto the 37°C-heated stage (Tokai Hit, Fujinomiya, Japan) of a Nikon TE 2000S inverted microscope. To improve image resolution, each oocyte was rotated by injection pipette to make the spindle clear in the equatorial plane of the oocyte. Karyoplasts enucleated from human oocytes were directly imaged. The length and width of the spindle in oocytes and karyoplasts were measured using ImageJ software (v1.8.0, National Institutes of Health, Bethesda, MD, USA). After spindle visualization, human oocytes and karyoplasts were ready for the ST or PNT procedure.

### Human spindle–chromosomal complex transfer

MII human oocytes (n = 30), obtained by IVM, from 15 donors (two oocytes from each donor) were allocated into 1 and 5 μg/ml CB groups for modified ST and ST, respectively. To avoid wasting human reproductive resources, each group of two oocytes served as a cytoplasmic recipient versus nuclear donor, reciprocally. The manipulations to prepare recipient cytoplasts, nuclear donor, ST and fertilization were performed according to the report from Mitalipov’s team (Tachibana *et al.*, 2013). Briefly, one recipient oocyte was fixed to the holding needle by applying a vacuum. Then the oocytes were rotated by holding a micropipette until the spindle was clearly visible under the Oosight imaging system. After a hole was drilled in zona pellucida by a laser in the 3 o’clock position, the injection micropipette was placed adjacent to the spindle and a vacuum applied to enucleate the karyoplasts containing the spindle. The karyoplasts were exposed briefly to HVJ-E and then transferred into the enucleated recipient oocyte. All manipulations were performed with Narishige micromanipulators on a 37°C-heated stage of a Nikon TE 2000S inverted microscope. All oocytes were washed several times in G-GAMETE for recovery and ICSI.

### Human pronucleus transfer

Zygotes (n = 30, developed from 38 MII human oocytes after IVM) from 15 donors were divided randomly into separate microdroplets containing different concentrations of CB (1 and 5 μg/ml). In accordance with ST, donor zygotes were used for PNT to exchange mitochondria genotypes. The manipulation of the enucleating pronuclei for recipient cytoplasts, aspiration of pronuclei for donor karyoplasts and PNT (including PNT and modified PNT) were performed as described by Herbert’s team (Hyslop *et al.*, 2016). Briefly, penetration of the zona pellucida of human zygotes was performed as mentioned in the ST manipulation. Then the double pronuclei of one zygote were enucleated and aspirated in a pipette with a diameter of 25 mm. After exposure to HVJ-E, donor pronuclei were transferred into enucleated recipient zygotes. The reconstructed zygotes were washed three times in G-1 PLUS medium, then were cultured in G-1 PLUS medium at 37°C, in a tabletop incubator for 120 h.

### Digital PCR for mtDNA copy number

The karyoplasts of spindle–chromosomal complex and pronuclei were isolated and lysed using a REPLI-g Single Cell Kit (150345, Qiagen, Hilden, Germany). The lysate of pronuclei was diluted 10 times before digital PCR (dPCR), while the lysate of the spindle–chromosomal complex was not diluted. A target template spanning nt16413–nt16481 of mtDNA was chosen, which was present only in mtDNA but not in nuclear DNA, and was verified in NCBI Primer BLAST. The primer sequences used were (5′ to 3′): GAAATCAATATCCCGCACAAGAG (forward) and TTAGCTACCCCCAAGTGTTATGG (reverse) with a TaqMan probe sequence of 5-FAM-ACTCTCCTCGCTCCGG-MGB-3 (Invitrogen, Thermo Fisher Scientific, Carlsbad, CA, USA) designed using Primer Express software (v3.0, Thermo Fisher Scientific, South San Francisco, California, USA). The quantification of mtDNA was performed as we described previously (Wang *et al.*, 2014). The sample was loaded using the QuantStudio 3D Digital PCR Chip Loader (4482592, Thermo Fisher Scientific, Singapore) and amplified in a GeneAmp PCR System 9700. Images of chips were taken using the QuantStudio 3D Digital PCR System (Thermo Fisher Scientific, Singapore) and analyzed with QuantStudio 3D AnalysisSuite software (v1.1, Thermo Fisher Scientific, Carlsbad, CA, USA).

### Pyrosequencing for mtDNA heteroplasmy

Heteroplasmy of donor mtDNA was evaluated by pyrosequencing. The region of the human mitochondrial displacement loop (D-loop) was amplified from the total genome using the following primers (5′ to 3′): TTAAACTATTCTCTGTTCTTTCATGGG (forward) and AAACATTTTCAGTGTATTGCTTTGAG (reverse) designed using NCBI Primer BLAST. The amplified D-loops were then cloned into the pMD18-T vector (6011, Takara, Japan) according to the manufacturer’s instructions. The plasmid with the PCR products was transformed into JM109 competent cells for propagating. The cloned PCR products were sequenced, and the informative single-nucleotide polymorphism (SNP) sites were identified using Sequencher (v4.7, Gene Codes Corporation, Ann Arbor, MI, USA). For detecting the donor mtDNA heteroplasmy in reconstituted embryos, pyrosequencing primers were designed using PyroMark Assay Design software (v2.0, Qiagen, Hilden, Germany), according to different SNP sites between donor and recipient woman. The SNP sites and primers are listed in Supplementary Table SII. The detection of mtDNA heteroplasmy was then performed as described previously (Wang *et al.*, 2014). Briefly, single-stranded biotinylated DNA was prepared from the PCR product with Pyrosequencing Vacuum Prep Tool (Biotage AB, Uppsala, Sweden). After mixing with 40 μl annealing buffer and 1.5 μl sequencing primer, samples were denaturated at 85°C for 2 min. Then pyrosequencing was performed on a PyroMark Q96 ID platform (Qiagen, Hilden, Germany). The mtDNA heteroplasmy level was determined by the allele quantification function mode of PyroMark Q96 software (v2.5.8.15, Qiagen, Hilden, Germany).

### Statistical analysis

All statistical analyses were performed using the Prism 8.0 statistical analysis program (GraphPad, San Diego, CA, USA). The Chi-squared test was used to assess mitochondrial distribution patterns. ANOVA was used to assess the oocyte and embryo development rates. The unpaired two-sided Student’s t-test was used to assess F-actin level, mitochondrial ultrastructure features, spindle length and width, and mtDNA copy number. The Mann–Whitney test was used to assess mtDNA heteroplasmy. Data were tested for normality before applying ANOVA, the unpaired two-sided Student’s t-test and the Mann–Whitney test using the Q–Q plot and Kolmogorov–Smirnov method. The significance level was set at *P *<* *0.05.

## Results

### Mitochondria tended to aggregate with an increase of CB concentration

**Figure 1.**
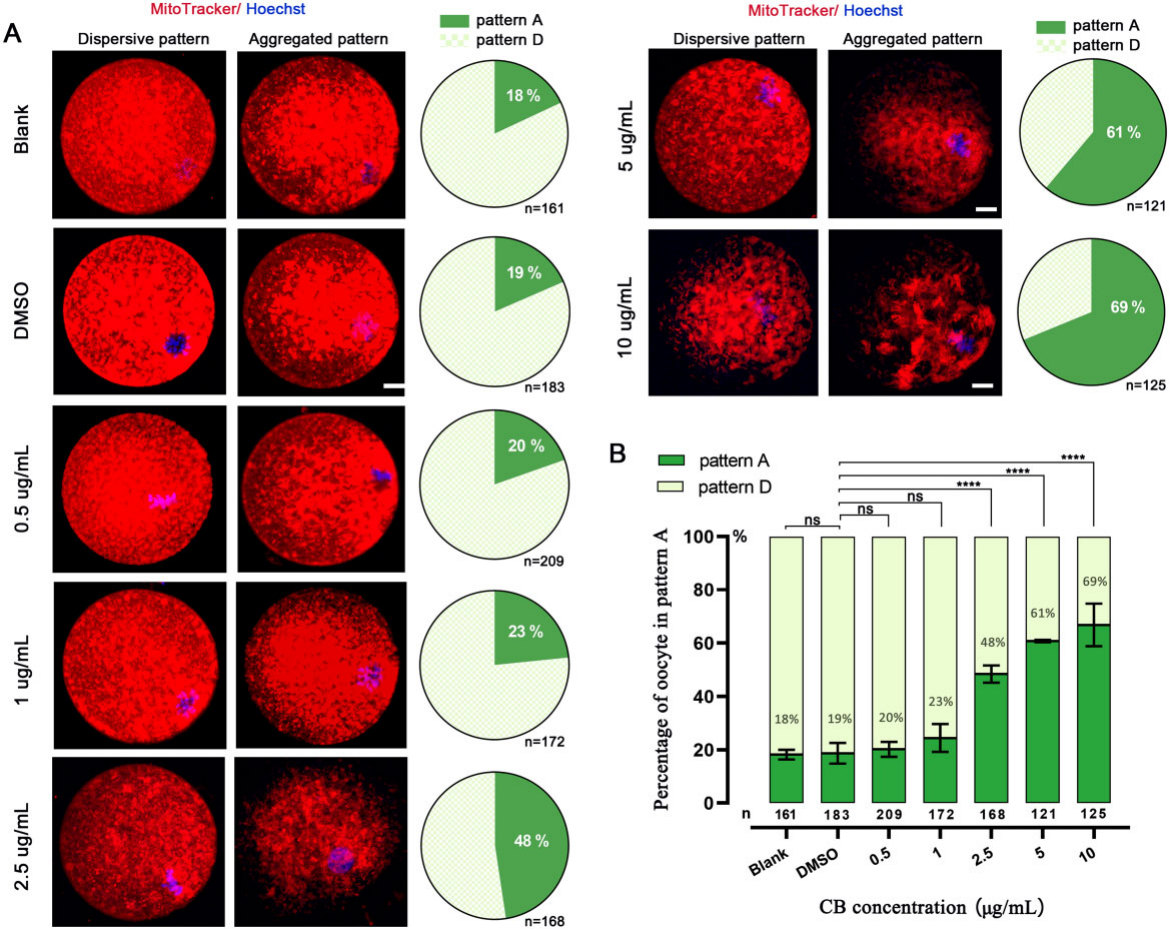
**Mitochondrial distribution in mouse oocytes treated with increasing concentrations of cytochalasin B.** (**A**) Mouse oocytes were stained for mitochondria (Mito Tracker, red) and chromosomes (Hoechst, blue) and imaged by confocal microscopy with projected z stacks. Dispersive pattern (Pattern D): mitochondria were distributed throughout the oocytes (left), aggregated pattern (Pattern A): the majority of mitochondria aggregated around the chromosomes (middle). Pie charts show the proportion of oocytes in pattern A in each group (right). Scale bar, 10 μm. (**B**) The percentage of oocytes in Pattern A when treated with different concentrations of cytochalasin B. Asterisks denote significant effects: ****P < 0.0001 (Chi-squared test), compared with the dimethyl sulfoxide (DMSO) group. Error bars indicate SD, with the mean value and number shown per group.

Two kinds of mitochondrial distribution patterns were observed in each group, namely a dispersive pattern (Pattern D), i.e. mitochondria were distributed diffusely throughout the oocyte (Fig. 1A, left), and an aggregated pattern (Pattern A), i.e. majority of mitochondria aggregated around the chromosomes (Fig. 1A, middle). Compared with the blank group, 0.1% DMSO had no effect on mitochondrial distribution in mouse oocytes. There were no significant differences in distribution patterns among the 1 µg/ml CB, 0.5 µg/ml CB and DMSO groups (23% versus 20% versus 19%, *P*>0.05) (Fig. 1B). However, the percentage of oocytes in Pattern A rose gradually with the increase of CB concentration from 2.5 to 10 µg/ml, compared with the DMSO group (48%, 61%, 69% versus 19%, respectively, *P* < 0.001) (Fig. 1B). Furthermore, we observed that mitochondria formed clusters and clumped into condensed masses as the CB concentration exceeded 2.5 μg/ml. These results indicated that low concentrations (0.5 or 1 μg/ml) of CB may be potential candidates, relative to the standard 5 µg/ml, for MRT.

### Higher CB concentrations affect the cytoskeleton in mouse oocytes

**Figure 2.**
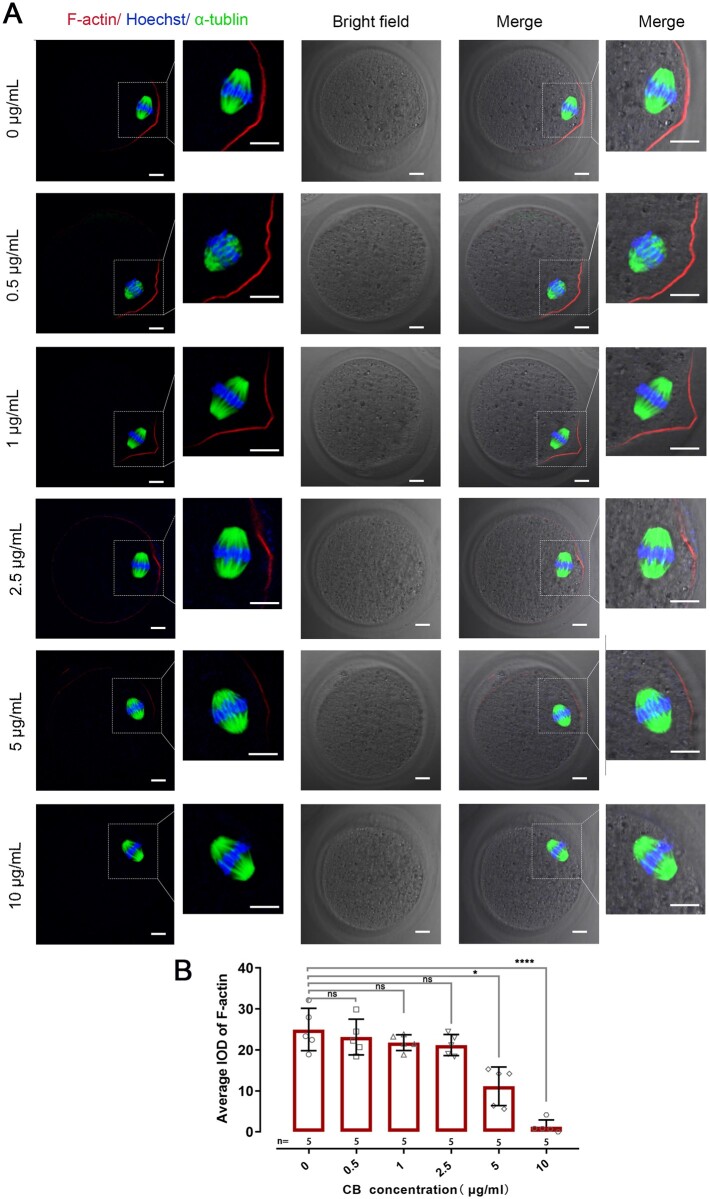
**Immunofluorescence staining of F-actin and α-tubulin in mouse oocytes treated with increasing concentrations of cytochalasin B.** (**A**) Representative confocal images of mouse metaphase II oocytes treated with different concentrations of cytochalasin B (CB). Images in second and far right columns are high-magnification views of the boxed area to the left. Oocytes were co-stained with antibodies to F-actin (red), α-tubulin (green) and Hoechst (blue). Bar = 10 μm. (**B**) Relative levels (average integrated optical density) of F-actin in the oocyte cortical domain treated with different concentrations of CB. Asterisks denote significant effects: *P < 0.05, ****P < 0.0001 (unpaired two-sided Student’s t-test), compared with 0 μg/ml group. Error bars indicate SD, with the mean value and n = 5 per group.

Both the cortical domain actin cap and the spindle, which are characterized by the enrichment of F-actin and tubulin, respectively, are two important cytoskeletons for meiosis. To observe whether high concentrations of CB affect the cytoskeleton, the actin-binding dye phalloidin, and α-tubulin were co-labeled to visualize the domain actin cap and spindle in mouse oocytes after treatment with different concentrations of CB (0, 0.5, 1, 2.5, 5 and 10 μg/ml CB group). We found that the actin cap gradually weakened and narrowed down as the concentration of CB gradient increased (Fig. 2A). The intensity of F-actin significantly decreased in the oocyte cortical domain in the 5 μg/ml CB group (n = 5, *P* < 0.05) and 10 μg/ml CB group (n = 5, *P* < 0.0001) when compared with the control group, while there were no significant differences between 0.5, 1 and 2.5 μg/ml CB and the control group (n = 5 in each group, *P*>0.05) (Fig. 2B).

α-tubulin staining showed that spindles transformed from an oval to a rectangular shape with an increase of CB concentration (Fig. 2A). Oocyte imaging showed no obvious difference in spindle length between each group treated with different concentrations of CB, while a significant difference occurred in spindle width. Compared with the control group, the width gradually increased with an increase of CB concentration (Supplementary Fig. S1A and B). Interestingly, the increased width in low CB concentration groups, including 0.5 and 1 μg/ml, could be partly reversed to a normal value after restoration in G-GAMETE medium *in vitro* for 0.5 h (Supplementary Fig. S1C and D). However, spindle width remained significantly wider in the 2.5 μg/ml group (9.4 ± 0.4 μm, n = 26) (*P* < 0.001) and 5 μg/ml group (9.5 ± 0.5 μm, n = 35) (*P* < 0.0001), compared with the control group (8.9 ± 0.5 μm, n = 49) after restoration for the same time *in vitro* (Supplementary Fig. S1D), indicating high CB concentration has an irreversible effect on the spindle width of mouse oocytes.

### The optimal concentration for mouse MRT is 1 μg/ml CB

To identify an optimal CB concentration for MRT, we further compared the effect of different CB concentration (0.5, 1, 2.5 and 5 μg/ml) on mouse MRT (ST and PNT) efficiency. As shown in Table I, there were no significant differences in fertilization and blastocyst formation rates between the four ST groups, except that 0.5 μg/ml CB resulted in a low survival rate of ST. We also observed similar results in PNT (Table II), suggesting that 1 μg/ml CB is the optimal concentration for MRT.

**Table I:** Effect of increasing concentrations of cytochalasin B on mouse spindle transfer efficiency and embryo development.

**Cytochalasin B concentration** **(μg/ml)**	Oocyte number	Survival (%)	Fertilization (%)	Blastocyst (%)
**0.5**	28	19 (67.9)	17 (89.5)	14 (82.4)
**1**	30	25 (83.3)	22 (88.0)	19 (86.4)
**2.5**	24	21 (87.5)	19 (90.5)	16 (84.2)
**5**	27	24 (88.9)	21 (87.5)	18 (85.7)

**Table II:** Effect of increasing concentrations of cytochalasin B on mouse pronuclear transfer efficiency and embryo development.

Cytochalasin B concentration (μg/ml)	Zygote number	Survival (%)	Cleavage (%)	Blastocyst (%)
**0.5**	23	16 (69.6)	16 (100)	13 (81.3)
**1**	27	22 (81.5)	22 (100)	18 (81.8)
**2.5**	24	20 (83.3)	20 (100)	16 (80.0)
**5**	26	22 (84.6)	22 (100)	18 (81.8)

### Mitochondrial ultrastructure in mouse oocytes was maintained in 1 μg/ml CB

**Figure 3:**
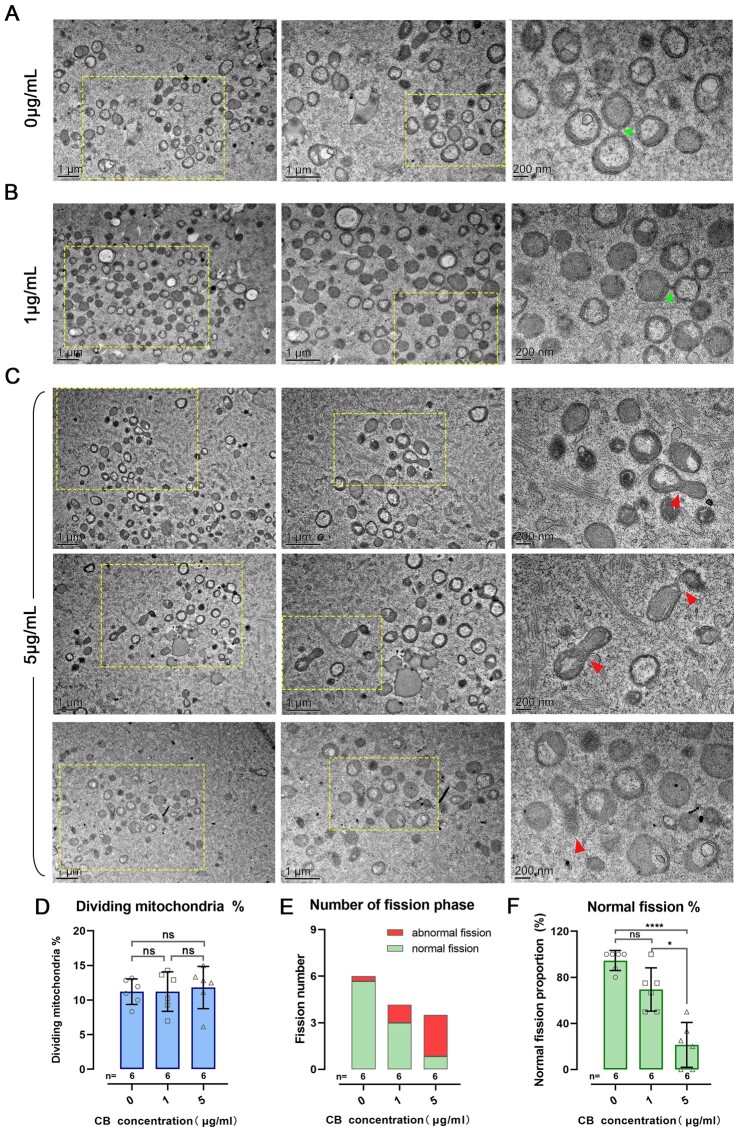
**Ultrastructural morphology of mouse oocytes treated with different concentrations of cytochalasin B.** (**A–C**) Ultrastructural morphology of mitochondria in mouse oocytes treated with 0, 1 and 5 μg/ml cytochalasin B (CB), respectively. Images in the middle column are high-magnification views of the boxed area on the left, images on the right are high-magnification views of the boxed area in the middle column. (**A** and **B**) Green arrows indicate normal mitochondrial fission that is about to complete. (**C**) Red arrowheads denote the abnormal and elongated mitochondria with swollen or transverse cristae. (**D**) Percentage of dividing mitochondria in each CB group. (**E**) The number of mitochondria undergoing abnormal and normal fission in each CB group. (**F**) Percentage of mitochondria undergoing abnormal fission (out of total fission) in each CB group. Asterisks denote significant effects: *P < 0.05, ****P < 0.0001 (unpaired two-sided Student’s t-test). Error bars indicate SD, with the mean value and n = 6 per group.

Since cytoskeletons can affect mitochondrial shape, CB treatment in MRT may influence mitochondrial morphology in mouse oocytes. TEM showed that mitochondrial morphologies in the 1 μg/ml group, similar to that in the control group, are typically spherical with few and truncated cristae surrounding a matrix of high-electron density, which indicated they were in a primitive state. Normal fission phases were observed in these two groups (Fig. 3A and B). Only a few mitochondria displayed electron-translucent spaces within the matrix. However, the mitochondria of oocytes from the 5 μg/ml group revealed more occurrences of electron-translucent spaces. Moreover, abnormal and elongated mitochondria occurred in oocytes treated in 5 μg/ml CB (Fig. 3C), suggesting a high concentration of CB might result in abnormal mitochondrial fission. Statistical analysis found that the mean proportions of dividing mitochondria in the three groups were similar (11.2% ± 1.8% versus 11.2% ± 2.9% versus 11.8% ± 3.0%, for control, 1 and 5 μg/ml groups, respectively, *P*>0.05) (Fig. 3D). The normal fission proportion (normal fission number/total fission number) in the 5 μg/ml group (21.4% ± 19.4%, n = 6) was obviously lower than in the 1 μg/ml group (69.4% ± 18.8%, n = 6) (*P* < 0.05) and in the control group (94.6% ± 8.7%, n = 6) (*P *<* *0.0001). Instead, no significant difference in normal fission proportion was found between the 1 μg/ml and control groups (69.4% ± 18.8% versus 94.6% ± 8.7%, n = 6) (*P*>0.05) (Fig. 3E and F). The results from TEM images indicated that 1 μg/ml CB has few adverse effects on mitochondrial ultrastructure, relative to the standard concentration of 5 μg/ml CB.

### CB at 1 μg/ml maintained the normal status of human spindles

We next compared the effect of two concentrations of CB (1 and 5 μg/ml) on the spindle status of human oocytes using Oosight imaging. There was no statistically significant difference in length or width between the 1 μg/ml group (12.2 ± 2.3 and 7.4 ± 1.0 μm, respectively, n = 14) and the control group (11.4 ± 1.6 and 7.1 ± 0.7 μm, respectively, n = 23) (Supplementary Fig. S2A, top and middle, Supplementary Fig. S2B; *P*>0.05). However, 5 μg/ml CB evidently reduced spindle length and width (9.3 ± 2.0 μm, 6.2 ± 1.2 μm, respectively, n = 9), compared with the control group (11.4 ± 1.6 μm, 7.1 ± 0.7 μm, respectively, n = 23) (Supplementary Fig. S2A, bottom, Supplementary Fig. S2B; *P *<* *0.01 for length, *P *<* *0.05 for width). We further compared spindle length and width in isolated karyoplasts from the 1 μg/ml and 5 μg/ml groups. Data analysis showed that spindle length in karyoplasts from the 5 μg/ml group (9.9 ± 2.6 μm, n = 26) is shorter than that from the 1 μg/ml group (12.4 ± 1.8 μm, n = 21) (Supplementary Fig. S2C and D, top, *P *<* *0.001), while no obvious difference was found in spindle width between the 5 μg/ml group (6.9 ± 1.8 μm, n = 26) and the 1 μg/ml group (7.6 ± 1.1 μm, n = 21) (Supplementary Fig. S2C and D, bottom, *P* > 0.05).

### CB at 1 μg/ml decreased mtDNA heteroplasmy in MRT human embryos

**Figure 4.**
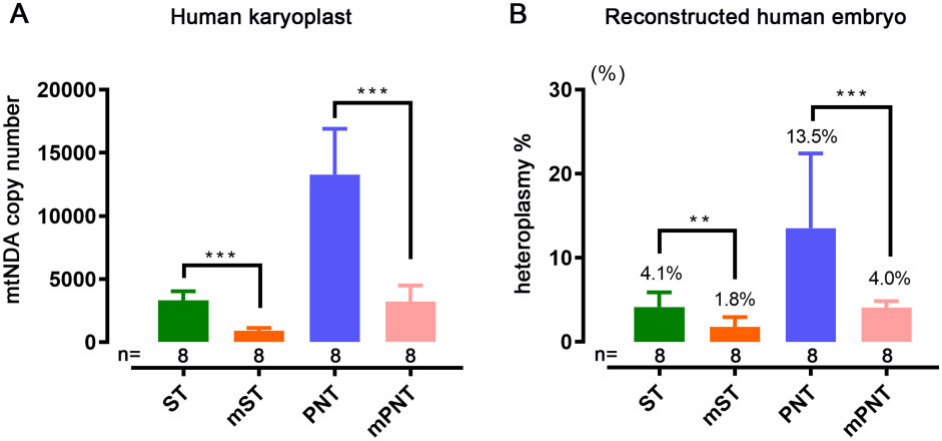
**Comparison of mitochondrial DNA (mtDNA) carryover after mitochondrial replacement therapies using the standard concentration of 5 μg/ml versus 1 μg/ml cytochalasin B.** (**A**) Quantification of human karyoplast mtDNA copy number by digital PCR. (**B**) Quantification by pyrosequencing of heteroplasmy in human reconstructed embryos after mitochondrial replacement therapies. The spindle transfer (ST) and pronuclear transfer (PNT) groups represent manipulation using the standard concentration of CB (5 μg/ml). The modified spindle transfer (mST) and modified pronuclear transfer (mPNT) groups represent manipulation using a modified CB concentration of 1 μg/ml. **P < 0.01, ***P < 0.001 (Mann–Whitney test). Error bars indicate SD, with the mean value and n = 8 per group.

To investigate whether 1 μg/ml CB instead of the standard 5 μg/ml CB could lower mtDNA heteroplasmy in MRT human embryos, we detected mtDNA carryover in isolated karyoplasts during human ST and PNT by applying 1 μg/ml CB and 5 μg/ml CB, respectively. dPCR analysis showed that karyoplasts from human MII oocytes in the 1 μg/ml group contained 888 molecules on average (n = 5), which was evidently lower than that in the 5 μg/ml group (3302 on average, n = 5) (*P *<* *0.0001). Similarly, the karyoplasts isolated from human zygotes also had a lower mtDNA copy number in the 1 μg/ml group (3024 on average, n = 5) than in the 5 μg/ml group (13 270 on average, n = 5) (*P *<* *0.0001) (Fig. 4A).

Next, the level of mtDNA heteroplasmy in the reconstructed embryos after MRT was measured by pyrosequencing. The mean heteroplasmy level for the ST-generated embryos in the 1 μg/ml group was 1.8% ± 1.1% (mean ± SD, n = 8), which was dramatically lower than the 5 μg/ml group (4.1% ± 1.8%, n = 8) (*P *<* *0.01). The PNT-generated embryos in the 1 μg/ml group had 4.0% ± 0.8% mtDNA carryover on average (n = 8), which was significantly fewer than in the 5 μg/ml group (13.5% ± 8.9%, n = 8) (*P *<* *0.001) (Fig. 4B). These data indicated that 1 μg/ml CB can reduce mtDNA heteroplasmy in the reconstructed embryos, while a concentration of 5 μg/ml CB failed to do so.

## Discussion

In the present study, we found that 1 μg/ml CB is the optimal concentration for MRT manipulation, evidenced by the character of mitochondrial distribution, mitochondrial and spindle morphology as well as the level of mtDNA heteroplasmy. This study is, to the best of our knowledge, the first to demonstrate that adjustment of CB concentration during enucleation might serve as a highly feasible and easy approach to improve the efficiency and safety of MRT.

Indubitably, mitochondrial distribution is responsible for the architecture and function of a cell. Changes in mitochondrial distribution have been mapped both in mouse and human oocytes during maturation. During mouse oogenesis, mitochondria aggregate around the nucleus at the GV and MI stage (Van Blerkom, 1991; Nishi *et al.*, 2003; Eichenlaub-Ritter *et al.*, 2011), while they dispersed homogenously at the first and second meiosis (Fulka, 2004). In humans, Takahashi *et al.* observed that mitochondria formed cluster-like aggregations at the GV stage, whereas they distributed evenly around the MI and MII period (Takahashi *et al.*, 2016). We found that two kinds of mitochondrial distribution patterns occurred in mouse MII oocytes, including an even distribution in cytoplasm and aggregation toward the nucleus. The ratio of the two distribution patterns in oocytes changed with the concentration of CB. The proportion of mouse MII oocytes in an aggregated pattern increased with increasing CB concentration. Our results were consistent with findings of Yu *et al.* regarding the dynamic observation of mitochondrial aggregating tendency in mouse oocytes (Yu *et al.*, 2010). Given the essential role of CB on F-actin during MRT, we speculated that microfilaments are important in the movement and localization of mitochondria inside the oocytes. We found that modifying the concentration of CB from 5 to 1 μg/ml could alleviate the aggregation and maintain the mitochondrial spatial distribution during MRT manipulation.

During meiotic maturation, asymmetric positioning of the meiotic spindle and polar body extrusion is driven by the dynamic changes of actin polymerization and depolymerization (Yi *et al.*, 2013). Thus, the actin cap, positioned above the oocyte spindle and characterized by enrichment of F-actin, is a key factor in meiosis (Deng *et al.*, 2007; Sun *et al.*, 2011; Yi *et al.*, 2011). Our data showed that the cortical actin cap of mouse oocytes diminished with an increase of CB concentration. When the CB concentration reached 10 μg/ml, the actin cap completely disappeared, consistent with the previous findings (Sun *et al.*, 2011). The irreversible change in spindle shape of both mouse and human oocytes suggested that a high concentration of CB (>2.5 μg/ml) inhibited actin polymerization in the oocyte and thus disturbed spindle organization.

Mitochondria are dynamic organelles whose shapes are controlled by precise regulation of fusion and fission (Karbowski and Youle, 2003). Balancing these two processes is necessary to maintain oocyte homeostasis and adjust mitochondrial function to meet the cellular needs (Chen and Chan, 2005; Wakai *et al.*, 2014). Breaking the balance can remarkably alter the mitochondrial morphology and disrupt the 3D network formed by actin filaments (Takahashi *et al.*, 2016). In our current work, the confocal imaging (using MitoTracker) in mouse oocytes showed that a mitochondrial ‘cloud’ occurred in the experiment groups treated with a high concentration of CB, especially in the 5 and 10 μg/ml groups (Fig. 1A). TEM results indicated that the phenomenon of the mitochondrial ‘cloud’ may be caused by an elongated mitochondrial shape, which are oval or round mitochondria in the control group. These findings are consistent with the results of recent studies showing that cyclic F-actin assembly on the outer mitochondrial membrane contributed to mitochondrial fission, and down-regulation of actin led to the elongation of mitochondria (Li *et al.*, 2015). In addition, recent research also reported that distinct fission signatures predict mitochondrial degradation or biogenesis; division at the periphery enables damaged mitochondria destined for mitophagy, whereas division at the midzone mediated by actin leads to proliferation of mitochondria (Kleele *et al.*, 2021). In combination with the experiments above, we proposed that high CB concentration may lead to abnormal mitochondrial fission and cellular dysfunction owing to an imbalance of the actin network. As mitochondrial fusion and fission were mainly conducted in somatic cell lines, the details of the interaction mechanism between the actin network and mitochondrial dynamics in oocytes remains unclear. Further study is needed to clarify this.

In summary, we conclude that CB plays an important role in mitochondrial redistribution and reshaping during oocyte maturation and embryo development. Our finding provides convincing evidence for optimizing CB concentration to minimize the mtDNA carryover during mitochondria replacement, therefore improving the efficiency and safety of MRT. More significantly, it has great potential for translational medicine in human reproduction, such as in the preparation of a defined medium for micromanipulation, benefiting patients with mitochondrial diseases as well as basic research on nuclear transfer in the future.

## Supplementary data

Supplementary data are available at *Molecular Human Reproduction* online.

## Supplementary Material

gaac036_Supplementary_Data

## Data Availability

The data underlying this article are available in the article and in its online supplementary material. Raw data will be shared on reasonable request to the corresponding authors.
