# Supplementary material for: Mitochondrial aggregation caused by cytochalasin B compromises the efficiency and safety of three-parent embryo
Source: Mol Hum Reprod. 2022 Oct 20;28(11):gaac036. doi: 10.1093/molehr/gaac036 (PMC9648687; doi:10.1093/molehr/gaac036)
Supplement: gaac036_Supplementary_Data [file gaac036_supplementary_data.pdf]

Mitochondrial aggregation caused by cytochalasin B compromises the efficiency and safety of three-parent embryo

Ying Li[1#], Sanbao Shi[2#], Jin Yuan[3,4#], Xi Xiao[1], Dongmei Ji[3], Jianxin Pan [2], Zhunyuan Min[2], Hao Wang[2], Hongying Sha[2]*, Yazhong Ji[1]*

[1]Reproductive Medicine Center, Tongji Hospital Affiliated to Tongji University, Shanghai 200065, China

[2]State Key Laboratory of Medical Neurobiology, Institutes of Brain Science, Shanghai Medical College, Fudan University, Shanghai 200032, China

[3]Center for Reproductive Medicine, Department of Obstetrics and Gynecology, the First Hospital Affiliated for Anhui Medical University, Hefei 230022, China

[4]The International Peace Maternal and Child Health Hospital, School of Medicine, Shanghai Jiao Tong University, Shanghai 200000, China

*****Correspondence should be addressed to Yazhong Ji or Hongying Sha; Email: jiyazhong@hotmail.com (Y.J.); Email: shahongying@fudan.edu.cn (H.S.)

[#] (Ying Li, Sanbao Shi and Jin Yuan contributed equally to this work)

Supplementary information

Supplementary Figure S1 Spindle morphology of mouse oocytes treated with increasing cytochalasin B concentrations.

Supplementary Figure S2 Spindle morphology of human oocytes and karyoplasts treated with different concentrations of cytochalasin B.

Supplementary Table SI The oocyte number of three biological replicates in each cytochalasin B group.

Supplementary Table SII Primer sequences of the specific single nucleotide polymorphisms between donors and recipient women.

Supplementary Figure S1

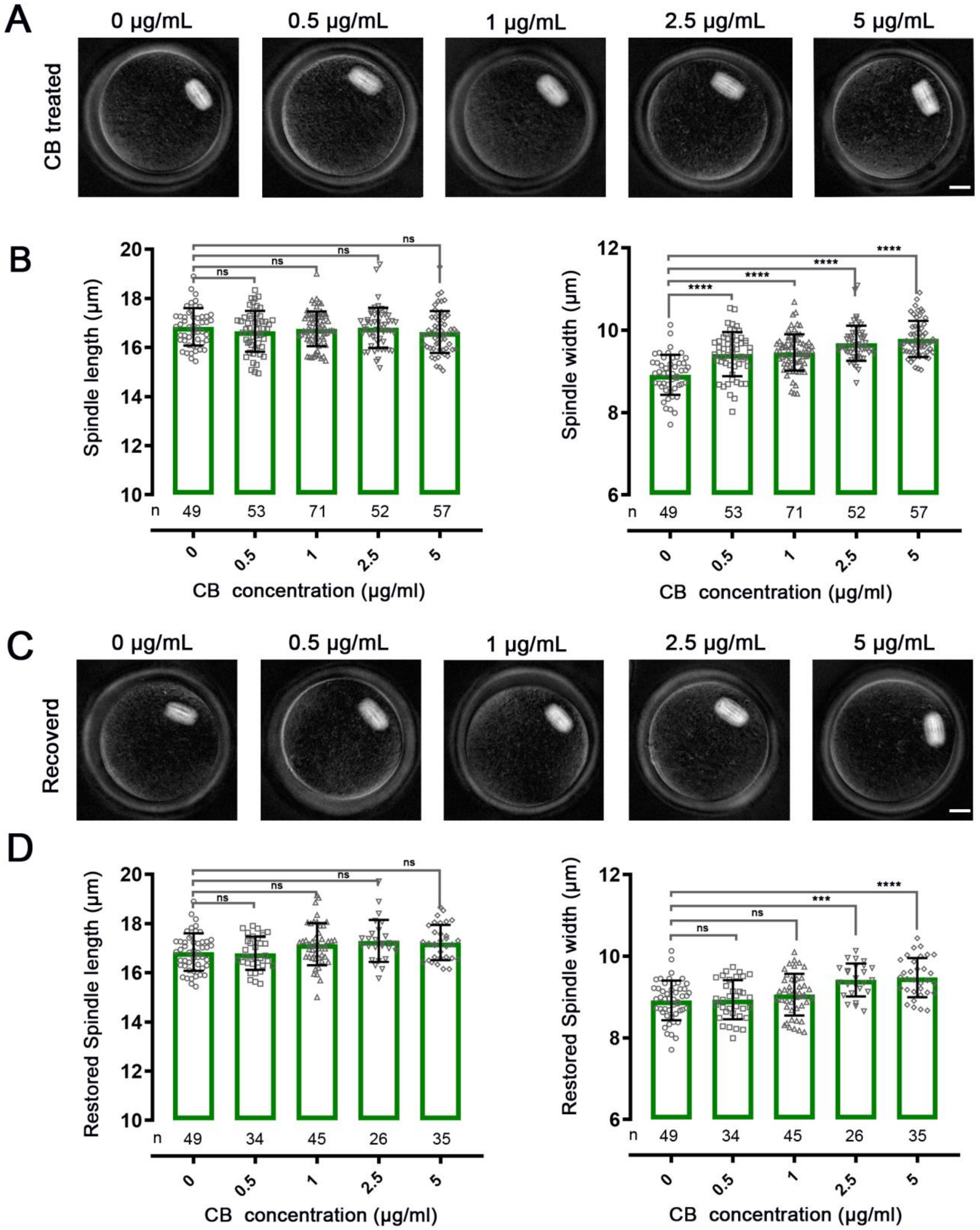

Supplementary Figure S1 Spindle morphology of mouse oocytes treated with increasing cytochalasin B concentrations. (A) Images of mouse MII oocytes treated with gradient concentrations of CB using Oosight Imaging System. Bar =10 μm. **(B)** Spindle length and width of mouse MII oocytes in each group cultured with different concentration of CB. **(C)** Images of restored mouse MII oocytes after treated with gradient concentrations of CB using Oosight Imaging System. Bar =10 μm. **(D)** Spindle length and width of restored mouse MII oocytes in each concentration of CB group. The data were presented as mean ±SD. $^{***}P < 0.001$, $^{****}P < 0.0001$, compared with the control group (0μg/mL).The number per group was shown below each column. CB, cytochalasin B.

Supplementary Figure S2

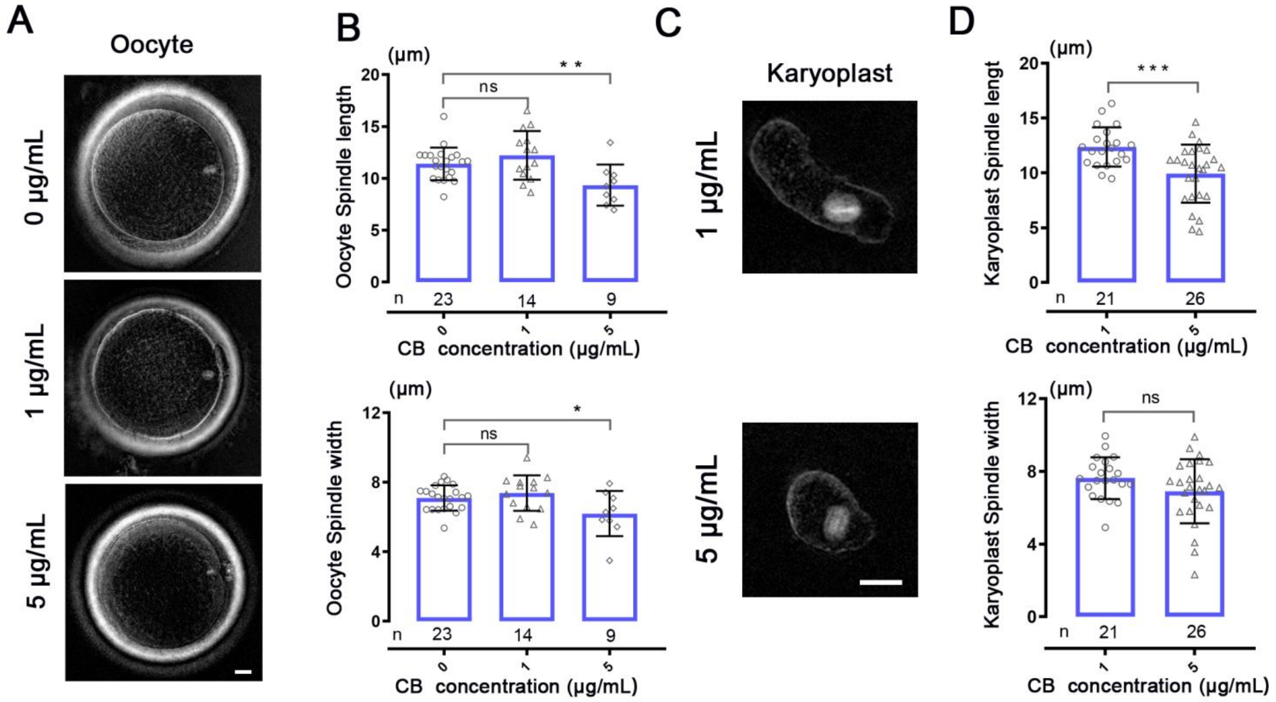

Supplementary Figure S2 Spindle morphology of human oocytes and karyoplasts treated with different concentrations of cytochalasin B. (A) Images of human MII oocytes using Oosight imaging system treated with 0 μg/ml (up), 1 μg/ml CB (middle) and 5 μg/ml CB (bottom). Bar = 10 μm. **(B)** Spindle length and width of human MII oocytes in each group cultured with different concentration of CB. **(C)** Images of human karyoplasts using Oosight imaging system cultured with 1 μg/mL CB (top) and 5 μg/ml CB (bottom). Bar = 10 μm. **(D)** Spindle length and width of human isolated karyoplasts in each concentration of CB group. The data were presented as mean ±SD. *$P < 0.05$, **$P < 0.01$, compared with the control group. The number per group was shown below each column. CB, cytochalasin B.

Supplementary Table SI. Oocyte number of three biological replicates in each cytochalasin B group.

Concentration of cytochalasin B (μg/mL)	D1 (n1)	D2 (n2)	D3 (n3)	Total (N)
**Blank**	68	45	48	**161**
**DMSO**	87	39	57	**183**
**0.5**	93	47	69	**209**
**1**	80	49	43	**172**
**2.5**	89	36	43	**168**
**5**	40	28	53	**121**
**10**	48	22	55	**125**
**Total oocyte number**	505	266	368	**1139**

Supplementary Table SII. Primer sequences of the specific single nucleotide polymorphisms between donors and recipient women.

Number	SNP	Primer sequence (5' to 3')
hmt-1	16051A/G	F: CTCTGTTCTTTCATGGGGAAGCA<br>R: TGGCTGGCAGTAATGTACGAAATA-5'Biotin<br>S: GGAAGCAGATTTGGGT
hmt-2	16129G/A	F: CTGCCAGCCACCATGAAT<br>R: GTGAGGGGTGGCTTTGGA-5'Biotin<br>S: AGCCACCATGAATATTG
hmt-3	16234C/T	F: CCCTCCCCATGCTTACAA<br>R: TGGCTTTGGGGTTGCAGT-5'Biotin<br>S: GCAATCAACCTTCAACTAT
hmt-4	16234C/T	F: CCCTCCCCATGCTTACAA<br>R: GGCGAGAAGGGATTTGACT-5'Biotin<br>S: GCAATCAACCTTCAACTAT
hmt-5	16319G/A	F: CACCCACTAGGATACCAACAAAC<br>R: CGAGAAGGGATTTGACTGTAATG-5'Biotin<br>S: AACAGTACATAGTACATAAA
hmt-6	223T/C	F: GTCGCAGTATCTGTCTTTGATTCC<br>R: CGGCTGTGCAGACATTCAATT-5'Biotin<br>S: AATTGTTATTATTATGTCCT
hmt-7	16304T/C	F: ACAAACCTACCCACCCTTAACA<br>R: GTCAAGGGACCCCTATCTGAG-5'Biotin<br>S: CCTACCCACCCTTAACA
hmt-8	16209T/C	F: CCCCTCCCCATGCTTACA<br>R: GTGAGGGGTGGCTTTGGAGT-5'Biotin<br>S: TGAAGGTTGATTGCTG

SNP, single nucleotide polymorphism
